# Sotrovimab: is it effective in early treatment of mild and moderate COVID-19 infections? A retrospective study

**DOI:** 10.1186/s43168-021-00104-8

**Published:** 2021-12-20

**Authors:** Ahmed Elesdoudy

**Affiliations:** Chest Diseases and Tuberculosis Department, Faculty of Medicine, Menoufia University-Yasin, Abdelghaffar street-Shebin Alkom, Menouf-Menoufia, Egypt

**Keywords:** COVID-19, Efficacy, Mild, Moderate, Sotrovimab

## Abstract

**Background:**

The monoclonal antibody sotrovimab is manufactured to antagonize many types of coronaviruses including the SARS-CoV-2. It is used mainly to treat mild and moderate COVID-19 infection and to prevent the progression of the disease from critical disease to severe.

**Objectives:**

To assess the effectiveness of sotrovimab in the early treatment of mild and moderate COVID-19 infections and prevention of disease progression to severe and critical disease.

**Methods:**

This study was performed on 220 outpatients who have already received sotrovimab in Obaidullah Hospital, United Arab Emirates. All patients underwent the following before receiving sotrovimab: routine laboratory studies (CBC, liver function tests, and kidney function tests) and other laboratory tests (C reactive protein (CRP), D dimer, and chest x-ray). All patients received sotrovimab in a dose of 500 mg once intravenous infusion over 30 min. All laboratory studies and CXR are repeated after 1 week of receiving the dose of sotrovimab.

**Results:**

The outcome was 43 patients deteriorated (19.5%) and 177 patients improved (80.5%). The progress of patients’ symptoms after receiving sotrovimab where the shortness of breath (SOB) deteriorated in 43 patients (19.5%) and improved in 177 patients (80.5%). The cough symptom deteriorated in 43 patients (19.5%), improved in 177 patients (80.5%). The progress of patients' radiology (chest x-ray) where it is deteriorated in 43 patients (19.5%) and improved in 177 patients (80.5%). The rate of hospitalization after receiving sotrovimab where 41 patients were hospitalized (18.6%) and 179 patients were not hospitalized (81.4%). There was a statistically significant difference before and after using sotrovimab in vital signs, inflammatory markers, kidney function tests, electrolytes, endocrine functions, and hepatic profile.

**Conclusion:**

Among adults with mild and moderate COVID-19, the use of sotrovimab significantly improved resolution of symptoms, outcome, radiology, or laboratory marker and decreased hospitalization. The findings support using sotrovimab in the early treatment of mild and moderate COVID-19. Wide-scale studies may be required for clarifying the effects of sotrovimab in the treatment of mild and moderate COVID-19 infections.

## Introduction

The COVID-19 outbreak has ended the life of more than 3 million people all over the world. In the USA, there were 2.5 million hospitalizations in autumn of 2020 and, in Jan 2021, COVID-19 patients occupied about 79% of ICU beds in the hospitals [1, 2]. At-highest risk of hospitalization or death includes elderly patients and patients with comorbid conditions, such as chronic kidney disease, obesity, chronic pulmonary diseases, and diabetes mellitus [3–7].

The monoclonal antibody sotrovimab is manufactured to antagonize many types of coronaviruses including the COVID-19. It is used mainly to treat mild and moderate COVID-19 infection and to prevent the progression of the disease from critical disease to severe [8]. We hypothesized that a monoclonal antibody that antagonizes all sarbecoviruses would target a highly conserved epitope that would be functionally known as COVID-19 evolves. Supporting this theory, the in vitro sotrovimab remains active against many COVID-19 strains firstly discovered in the UK (B.1.1.7), South Africa (B.1.351), Brazil (P.1), and California (B.1.427/B.1.429) [9]. On the other hand, monoclonal anti-bodies manufactured for COVID-19 adhere to the receptor-binding element that includes the angiotensin-converting enzyme 2 (ACE2) receptor that characterizes by its mutating and immunological activity [10].

The aim of the work was the assessment of the effectiveness of sotrovimab in the early treatment of mild and moderate COVID-19 infections and prevention of disease progression to severe and critical disease.

## Methods

This retrospective study was done in Ibrahim Bin Hamad and Obaidullah Geriatric Hospitals, Ministry of Health, United Arab Emirates, from May 2021 to September 2021. The study was performed on 220 outpatients who have already received sotrovimab. The data were collected retrospectively from the hospital filing system. Approval of the ethical committee of the hospital had already been taken. All patients underwent the following before receiving the dose of sotrovimab: careful history taking, general and local examination, routine laboratory studies (CBC, liver function tests, kidney function tests), and other laboratory tests (C reactive protein (CRP), D dimer, and chest x-ray). All patients received sotrovimab in a dose of 500 mg once intravenous infusion over 30 min. All laboratory studies and CXR are repeated after 1 week of receiving the dose of sotrovimab. The laboratory test was done using reagents from Siemens company, Germany. The device used for CBC was ADVIA 560, Hematology System, Siemens Company, Germany. Clinical Inclusion Criteria: at high-risk patients for progressing to severe COVID-19 and/or hospitalization. Treatment began early after receiving a positive test on a COVID-19 testing and during 10 days of the onset of the symptoms. High-risk individuals as specified who meet at least one of the following: age ≥ 65 years, obesity (BMI ≥ 25 kg/m^2^), diabetes mellitus, cardiovascular disease (as well as hereditary cardiac diseases) or hypertension, chronic pulmonary diseases (e.g., COPD, moderate-to-severe asthma, pulmonary interstitial diseases, mucoviscidosis), an immuno-compromising condition or immunosuppressive treatment, long-standing renal impairment, pregnant women, sickle cell disease, and neuro-developmental diseases (e.g., CP). Clinical Exclusion Criteria: admitted patients because of COVID-19, patients who need O_2_ treatment because of COVID-19, and/or need of increasing in basal O_2_ flow-rate because of COVID-19.

### SARS-CoV-2 illness severity index

#### Asymptomatic or presymptomatic infection

Individuals who test positive for SARS-CoV-2 using a virologic test (i.e., a nucleic acid amplification test or an antigen test), but who have no symptoms that are consistent with COVID-19. *Mild illness*: Individuals who have any of the various signs and symptoms of COVID-19 (e.g., fever, cough, sore throat, malaise, headache, muscle pain) without shortness of breath, dyspnea, or abnormal chest imaging. *Moderate illness*: Individuals who have evidence of lower respiratory disease by clinical assessment or imaging, and a saturation of oxygen (SpO_2_) ≥94% on room air at sea level. *Severe illness*: Individuals who have respiratory frequency >30 breaths per minute, SpO_2_ 3%), ratio of arterial partial pressure of oxygen to fraction of inspired oxygen (PaO_2_/FiO_2_) 50%. *Critical illness*: Individuals who have respiratory failure, septic shock, and/or multiple organ dysfunction.

## Results

This retrospective study was done in Ibrahim Bin Hamad and Obaidullah Geriatric Hospitals, Ministry of Health, United Arab Emirates. The study was performed on 220 outpatients who have already received sotrovimab.

This study showed that the age range of the studied group was between 40 years and 107 years with mean age of 67.1±11.48. The sex distribution of the studied group was 124 male (56.4%) and 96 female (43.6%). The outcome of the studied group was 43 patients deteriorated (19.5%) and 177 patients improved (80.5%). This study showed the progress of patients’ symptoms after receiving sotrovimab where the shortness of breath (SOB) deteriorated in 43 patients (19.5%) and improved in 177 patients (80.5%). The cough symptom deteriorated in 43 patients (19.5%) and improved in 177 patients (80.5%). The progress of patients’ radiology (chest x-ray) where it is deteriorated in 43 patients (19.5%) and improved in 177 patients (80.5%). The study showed the rate of hospitalization after receiving sotrovimab where 41 patients were hospitalized (18.6%) and 179 patients were not hospitalized (81.4%). The associated co-morbidities: 18 patients had bronchial asthma (8.2%), 36 patients had chronic kidney disease (CKD) (16.4%), 91 patients had diabetes mellitus (DM) (41.4%), 99 patients had hypertension (HTN) (45%), 10 patient had hypothyroidism (4.5%), and 39 patients had ischemic heart diseases (IHD) (17.7%) (Table 1).

**Table 1:** Age, sex distribution, outcome, comorbidities, symptoms, and chest x-ray of the studied group

	Frequency	Percent
**Sex**		
**Male**	124	56.4
**Female**	96	43.6
**Age**		
**Mean±SD**	67.1±11.48	
**Median**	68	
**Range**	40–107	
**Outcomes**		
**Improved**	177	80.5
**Deteriorated**	43	19.5
**SOB after sotrovimab**		
**Improved**	177	80.5
**Deteriorated**	43	19.5
**Cough after sotrovimab**		
**Improved**	177	80.5
**Deteriorated**	43	19.5
**CXR after sotrovimab**		
**Improved**	177	80.5
**Deteriorated**	43	19.5
**Hospitalization after sotrovimab (primary outcome)**		
**Hospitalized**	41	18.6
**Not hospitalized**	179	81.4
**Comorbidities**		
**DM**		
**No**	129	58.6
**Yes**	91	41.4
**HTN**		
**No**	121	55.0
**Yes**	99	45.0
**CKD**		
**No**	184	83.6
**Yes**	36	16.4
**Asthma**		
**No**	202	91.8
**Yes**	18	8.2
**Hypothyroidism**		
**No**	210	95.5
**Yes**	10	4.5
**IHD**		
**No**	181	82.3
**Yes**	39	17.7

*SD* standard deviation

The study also showed that there was significant difference before and after using sotrovimab in vital signs: temprature (*P* value: 0.001), pulse (*P* value: 0.001), and respiratory rate (*P* value: 0.001). There was statistical significant difference in inflammatory markers: WBCs count (*P* value: 0.001), lymphocytes count (*P* value: 0.001), C-reactive pr0tein (CRP) (*P* value: 0.009), and D-dimer (*P* value:0.001). There was statistical significant difference in kidney function tests and electrolytes: urea (*P* value: 0.001), creatinine (*P* value: 0.001), GFR (*P* value: 0.001), Na (*P* value: 0.001), K (*P* value: 0.001), Ca (*P* value: 0.001), Mg (*P* value: 0.001), and phosphorous (*P* value: 0.001). There was statistical significant difference in endocrine functions: glucose (*P* value: 0.001), TSH (*P* value: 0.001), and free t4 (*P* value: 0.006). There was statistical significant difference in hepatic profile: ALT (*P* value: 0.001), AST (*P* value: 0.001), and serum albumin (*P* value: 0.001) (Table 2).

**Table 2:** Vital signs and laboratory studies of the studied group before and after using sotrovimab

	Before sotrovimab	After sotrovimab	Paired ***t*** test	***p*** value
**Temperature**	38.008±0.886	37.633±0.855	4.504	0.001^*^
**Pulse**	78.714±16.573	73.168±12.419	3.739	0.001^*^
**RR**	24.196±7.881	21.050±6.421	4.287	0.001^*^
**WBCs**	5626.818±2343.335	6249.546±2440.329	− 4.104	0.001^*^
**Lymphocytes**	1.829±0.740	2.083±0.801	− 3.709	0.001^*^
**CRP**	52.885±47.854	43.255±56.209	2.621	0.009^*^
**D-dimer**	1.880±1.611	2.956±4.097	− 3.750	0.001^*^
**UREA**	9.291±4.225	4.532±2.307	16.716	0.001^*^
**Creatinine**	158.591±95.976	68.314±19.915	14.137	0.001^*^
**GFR**	56.655±25.876	96.350±15.764	− 22.200	0.001^*^
**NA**	144.150±5.397	139.027±4.092	12.905	0.001^*^
**K**	4.804±1.198	4.219±0.258	6.857	0.001^*^
**Ca**	1.930±0.166	2.340±0.145	− 30.727	0.001^*^
**Mg**	0.841±0.102	1.006±0.066	− 29.099	0.001^*^
**Phosphorus**	0.903±0.171	1.106±0.217	− 22.069	0.001^*^
**Glucose**	13.226±3.966	9.091±2.142	13.751	0.001^*^
**TSH**	2.275±1.536	2.020±1.545	3.999	0.001^*^
**Free t4**	14.662±2.214	14.282±2.792	2.767	0.006^*^
**ALT**	113.232±102.281	66.377±61.647	5.791	0.001^*^
**AST**	73.723±64.139	40.518±18.064	7.283	0.001^*^
**Albumin**	19.446±4.848	33.523±6.588	− 32.492	0.001^*^

*SD* standard deviation

^*^Significant

## Discussion

This study showed the age of the group is ranging from 40 years to 107 years with mean age of 67.1±11.48. The sex distribution of the studied group was 124 male (56.4%) and 96 female (43.6%). The outcome of the studied group was 43 patients deteriorated (19.5%), 177 patients improved (80.5%). The study showed the rate of hospitalization after receiving sotrovimab where 41 patients were hospitalized (18.6%) and 179 patients were not hospitalized (81.4%).

This result coincides with the study done by Anil Gupta et al. [11]. It included 583 treated COVID-19 patients (sotrovimab, 291; placebo, 292); the main outcome endpoint was met. The progression of COVID-19 was markedly decreased by 85% (97.24% interval of confidence) and 3 (1%) patients progressed to the main outcome end-point in the sotrovimab group versus twenty-one (7%) patients in the group receiving placebo. The 5 patients admitted in ICU received placebo included a patient that passed away by the 29th day.

This also agrees with the trial of John Paul Verderese et al. [12] in which 707 confirmed COVID-19 patients received NmAb. Subjects who received the infusion monoclonal anti-body had markedly lower hospital admission rate (5.8% vs. 11.4%), a short stay length if admitted to hospital (mean 5.2 days vs. 7.4 days), and few emergency department visits during thirty days post index (8.1% vs 12.3%) than control.

This study showed the progress of patients’ symptoms after receiving sotrovimab where the shortness of breath (SOB) deteriorated in 43 patients (19.5%) and improved in 177 patients (80.5%). The cough symptom deteriorated in 43 patients (19.5%) and improved in 177 patients (80.5%). The progress of patients’ radiology (chest x-ray) where it is deteriorated in 43 patients (19.5%) and improved in 177 patients (80.5%).

Aeron C Hurt et al. [13] stated that the effectiveness of mAbs in subjects with COVID-19 that are hospitalized varies, having the potential to highlight the challenge of antiviral medications in subjects that went to severe disease. On the other hand, early data suggest an encouraging role for mAbs that provide effective COVID-19 prophylaxis.

Peter Chen et al. [14] performed a study for assessment of the efficacy of mAbs on COVID-19 symptoms. He compared the changes from basal symptoms score between the LY-CoV-555 group and the group of placebo. The symptoms score ranges from zero to twenty-four and include 8 domain graded from zero (no symptoms) to three (severe symptoms). From day two to day six, the changes in the symptoms score from baseline was best in the group of LY-CoV-555 than in the group of placebo, with values of − 0.79 (95% CI, −1.35 to −0.24) on day two, −0.57 (95% CI, −1.12 to −0.01) on day three, −1.04 (95% CI, −1.60 to −0.49) on day four, −0.73 (95% CI, −1.28 to −0.17) on day five, and −0.79 (95% CI, −1.35 to −0.23) on day six. The changes from basal line in the symptoms score continue to be best in the group of LY-CoV-555 than in the group of placebo from day seven to day eleven, though by these time-points many subjects in the 2 groups had recovered completely and/or had very mild symptoms.

In this study, in the associated co-morbidities, 18 patients had bronchial asthma (8.2%), 36 patients had chronic kidney disease (CKD) (16.4%), 91 patients had diabetes mellitus (DM) (41.4%), 99 patients had hypertension (HTN) (45%), 10 patient had hypothyroidism(4.5%), and 39 patient had ischemic heart diseases (IHD) (17.7%).

In the trial of Ravindra Ganesh et al. [15], the group consisted of 3596 subjects; median age was 62 years; and 50% were female. All had more than one medical co-morbidity; 55% had multiple co-morbidities. All cause- and COVID-19-related hospital admission rates at the 28th day were 3.98% and 2.56%, consecutively. There was no significant difference in all cause- and COVID-19-related hospital admission rates after receiving the mAbs (adjusted HR, 1.4, 95% CI 0.9–2.2 and 1.6, 95% CI 0.8–2.7, consecutively).

Preeti N. Malani et al. [16] stated that the Food and Drug Administration (USA) has declared Emergency Use Authorizations for mAbs including sotrovimab for out-patients with mild to moderate symptoms of COVID-19 and risk factors to progress into severe disease (e.g., old age, obese patients, DM, chronic renal impairment, and immunosuppressed patients).

The study also showed that there was a statistically significant difference before and after using sotrovimab in vital signs: temperature, pulse, and respiratory rate. There was a statistically significant difference in inflammatory markers: WBCs count, lymphocytes count, C-reactive protein (CRP), and D-dimer. There was a statistically significant difference in kidney function tests and electrolytes: urea, creatinine, GFR, Na, K, Ca, Mg, and phosphorous. There was a statistically significant difference in endocrine functions: Glucose, TSH, free t4. There was a statistically significant difference in hepatic profile: ALT, AST, and serum albumin.

These data showed that there was an improvement in clinical and laboratory markers after using sotrovimab. Actually, there was not enough data or literature regarding the laboratory markers before and after using sotrovimab except for the reduction of viral load.

The Food and Drug Administration (USA) [17] authorizes an emergency use (EUA) for the investigational monoclonal anti-body (sotrovimab) for the treatment of mild to moderate COVID-19 in adult patients and children (equal or more than 12 years, equal or more than 40 kg) and have positive COVID-19 test and at-high-risk to progress into severe COVID-19. This involves, e.g., patients with more than 65 years or patients with specific comorbidities. Sotrovimab is not authorized to subjects that are hospital admitted because of COVID-19 and/or need O_2_ treatment because of COVID-19. Sotrovimab does not show benefits in subjects that are hospital admitted because of COVID-19 and mAbs may be combined with the deterioration of the patient condition when given to hospital admitted subjects in need of high O_2_ flow and/or mechanical ventilation.

The European Medicines Agency (https://www.ema.europa.eu/en/documents/referral/sotrovimab-also-known-vir-7831-gsk4182136-covid19-article-53-procedure-assessment-report_en.pdf) starts a rolling review into VIR-7831 based on preliminary results from an ongoing study looking at the ability of the medicine to prevent hospitalization or death in non-hospitalized patients with COVID-19 (7 May 2021); an interim analysis of data from 583 patients enrolled in the COMET-ICE trial demonstrated an 85% (*p*=0.002) reduction in hospitalization or death in patients receiving VIR-7831 as monotherapy compared to placebo (10 March 2021).

## Conclusion

Among adults with mild and moderate COVID-19, the use of sotrovimab significantly improved resolution of symptoms, outcome, radiology, or laboratory marker and decreased the rate of hospitalization. The findings support using sotrovimab in early treatment of mild and moderate COVID-19. Wide-scale studies may be required for clarifying the effects of sotrovimab in the treatment of mild and moderate COVID-19 infections.

## Data Availability

The data that support the findings of this study are available from Ibrahim Bin Hamad Hospital, UAE, but restrictions apply to the availability of these data, which were used under license for the current study, and so are not publicly available. Data are however available from the authors upon reasonable request and with permission of Ibrahim Bin Hamad Hospital, UAE.
